# HIV Risk and the Alcohol Environment: Advancing an Ecological Epidemiology for HIV/AIDS

**Published:** 2010

**Authors:** Richard Scribner, Katherine P. Theall, Neal Simonsen, William Robinson

**Keywords:** Human immunodeficiency virus, acquired immune deficiency syndrome, risk factors, alcohol consumption, alcoholic beverage sales outlet, environmental factors, epidemiology, ecological epidemiology, high risk sexual behavior

## Abstract

The study of individual risk factors is inadequate to address the current public health challenges associated with HIV/AIDS. Rather, an ecological epidemiological study of HIV/AIDS is needed to address these challenges. A socioecological framework has been proposed for HIV/AIDS, including influences at the individual level, the interpersonal level, the neighborhood level, and the societal level. This framework provides the basis for a conceptual model with specific risk factors at each of these levels and cross-level associations. The nature of the associations also is important, in particular the assumption that the neighborhood alcohol environment exerts its effect on HIV risk through both direct and indirect pathways.

It has been widely recognized that an epidemiological study limited to individual risk factors will not be adequate to address the current public health challenges of human immunodeficiency virus (HIV)/acquired immune deficiency syndrome (AIDS) (Diez-Roux 1998; Susser 2004; Williams and Jackson 2005; Yen and Syme 1999). HIV, alcohol abuse and dependence, and health inequalities all involve complex interactions between social and biological factors. Over the past several decades, a new epidemiological approach, termed ecological epidemiology, has emerged to address these complexities. This article describes the current research associated with the development of an ecological epidemiology of HIV risk as it applies to the alcohol environment. It first presents an ecological framework for HIV risk. By presenting a multilevel structure in which social contexts are thought to influence health outcomes, ecological frameworks symbolize the conceptual break from risk factor epidemiology to ecological epidemiology that has occurred over the past decades. The article then introduces a conceptual model that describes relationships between selected risk factors that characterize the various exposures associated with social contexts.

## Ecological Epidemiology

**Figure 1:**
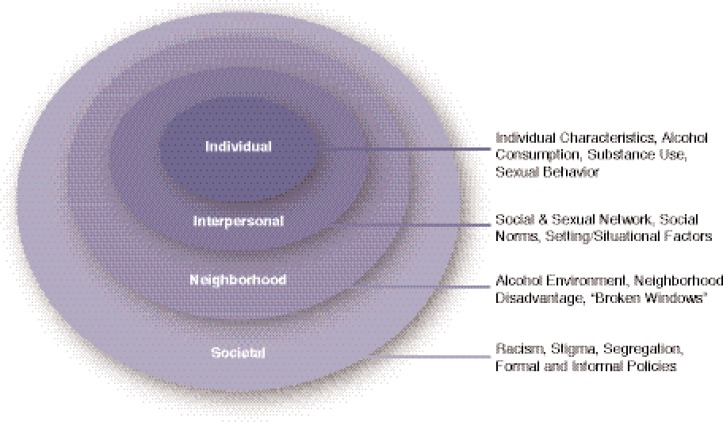
Socioecological framework for HIV/AIDS risk.

March and Susser (2006) originated the term ecological epidemiology, an approach to complex disease systems that characterizes disease risk in terms of multiple causative factors contributing in both social and physical contexts at the population level. Their insights inspired a number of researchers to propose ecological frameworks for a variety of diseases, including sexually transmitted infections (STIs) (Dahlberg and Krug 2002; DiClemente et al. 2005). These frameworks build on each other and typically include multiple levels of influence portrayed as concentric circles typically beginning with the individual level, followed by the interpersonal level, the neighborhood or community level, and finally the societal level. (Bronfenbrenner 1979; DiClemente et al. 2005) (see figure 1). These frameworks are based on the idea is that individuals operate within spheres of social influence and therefore prevention strategies should include a continuum of activities that address multiple spheres of influence. Frameworks of this type are generic and therefore do not provide measurable constructs that characterize social contexts and are believed to influence a particular health outcome. The conceptual model described below is designed to make explicit both the constructs that characterize the various spheres of social influence (i.e., social contexts) and the hypothetical relationships between them as they relate to alcohol and HIV risk so that hypotheses can be tested. In a sense, this represents the beginning of an ecological epidemiology of HIV.

The idea of an ecological epidemiology of HIV implies that there are measurable constructs at each of the levels of influence and that cross-level connections are involved in the occurrence of HIV, both at the individual (e.g., infection and transmission) and population (e.g., neighborhood HIV rates) levels. Statistically, this implies a multilevel framework in which connections between constructs at different levels are modeled as cross-level interactions. This article highlights the empirical research that has been conducted, which provides evidence for each of the connections and the areas in which future research is needed.

### Macrosocial and Microsocial Factors

The multilevel framework does not hold that social and environmental constructs are directly related to health outcomes. Rather, as proposed by Glass and McAtee (2006), a different dynamic is thought to operate when contextual factors influence individual level risk. They argue that macrosocial (e.g., State policies, neighborhood environments) and microsocial (e.g., social networks, social norms) factors can either constrain or promote the occurrence of individual-level behaviors associated with risk. According to Glass and McAtee (2006), macrosocial and microsocial factors can be thought of as risk regulators interacting with the individual-level factors to make certain outcomes more or less likely among individuals exposed to a particular social or environmental context. Their model also considers these relationships over the life course and the fact that relationships may be reciprocal. It should be noted that the interaction between risk regulators and the individual-level risk factors can be modeled statistically as a cross-level interaction in a multilevel model (e.g., variable slope).

## Conceptual Model

**Figure 2:**
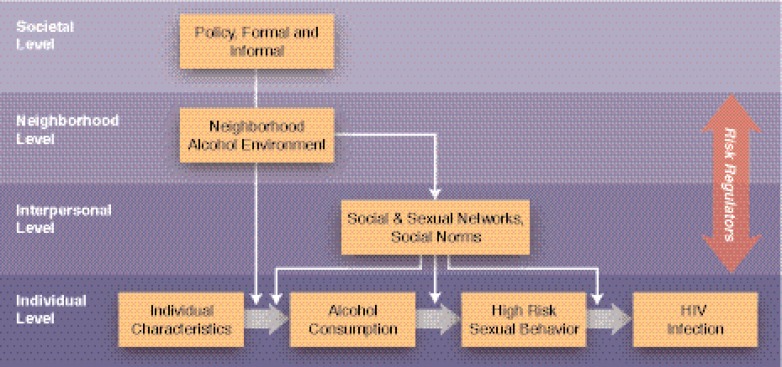
Conceptual model of HIV risk from an ecological perspective characterizing the role of the alcohol environment.

The authors’ conceptual model of HIV risk within an alcohol environment begins at the individual level, continues though the interpersonal level into the neighborhood level, and finishes at the societal level. It is a spatial hierarchy in which individuals are nested within interpersonal networks and neighborhoods, which are nested within societal structures (e.g., cities). Interpersonal social networks and neighborhoods tend to be misaligned with regard to this spatial hierarchy. For example, several studies have geo-referenced sexual networks, and the geographic distribution typically varies with the definition of the relationship (e.g., sexual, drug, social) (Rothenberg et al. 2005). On the other hand, social norms can either be very small, pertaining to a particular alcohol establishment, or very large, encompassing an entire society. In any case, in the authors’ model there is no hierarchical distinction between the interpersonal and neighborhood levels (see figure 2).

### Individual Level

An ecological epidemiology of HIV risk implies a multilevel structure, with individual-level risk factors at the base. This discussion specifically relates to those factors that appear to be affected by the neighborhood alcohol environment: alcohol consumption and high-risk sexual behaviors (e.g., sex without a condom, multiple sex partners, and concurrent partnerships). In the model, HIV infection is the final impact of these behaviors. For simplicity, the model does not include the links between the alcohol environment and intravenous drug use and other individual factors (e.g., personality, perceived risk, condom self-efficacy) that also play a role in these complex relationships.

### Interpersonal Level

The interpersonal level refers to the social and sexual networks in which individuals are imbedded. Considerable evidence exists to suggest that network structure determines the propagation of STIs like HIV and that position in the network, in terms of embedded-ness, is associated with individual risk (Aral 1999; Fichtenberg et al. 2009; Potterat et al. 2002). Sexual network factors that influence disease transmission include structural factors, such as network size, density, mixing, and turnover as well as compositional factors, such as network member characteristics (Fichtenberg et al. 2009).

The interpersonal level in this model encompasses the role of alcohol outlets in shaping social and sexual networks. Members of a sexual network, especially core group members, tend to be geographically clustered. Estimates of the median distance between partners in a network range from 0.34 km to 1.3 km, depending on the study, which emphasizes the importance of the local neighborhood in STI transmission dynamics (Rothenberg et al. 2005; Zenilman et al. 1999). Scribner and colleagues (2008) suggest that this geographic clustering of sexual network members can be used to locate the core group members using geo-referenced surveillance data and specialized spatial techniques. In addition, alcohol outlets tend to occupy central positions within sexual and social networks (De et al. 2004; Morris et al. 2008). However, nowhere is the relationship between alcohol outlets and networks more important than among men who have sex with men (MSM), a primary risk group for HIV. Gay bars, institutions within the MSM community (Achilles 1967), are among the few settings where the community can experience social interactions that are shaped by the social norms which tend to maintain the social and sexual networks defining a community.

### Neighborhood Level

The neighborhood level includes factors that define the neighborhood alcohol environment (e.g., density of alcohol outlets). According to the conceptual model presented here, the neighborhood alcohol environment influences HIV risk along two separate pathways. First, alcohol outlets, including both on-premise outlets such as bars and clubs and off-premise outlets such as liquor stores, affect risk of HIV and other STIs through their moderating effect on alcohol consumption. Higher densities of alcohol outlets have been geographically associated with increased consumption of alcohol at the neighborhood level (Gruenewald et al. 1993; Scribner et al. 2000, 2008; Treno et al. 2003), which in turn tends to influence the frequency of high-risk sexual behavior as well as risks associated with drug use. Second, the neighborhood alcohol environment may influence HIV risk through an indirect effect on social and sexual networks. As mentioned above, alcohol outlets often are important neighborhood locations for social and sexual networks. The density of alcohol outlets in a neighborhood affects social capital presumably by expanding or contracting social networks (Scribner et al. 2007; Theall et al. 2009). For example, a poorly run alcohol outlet that becomes a focus of criminal activity in a neighborhood will serve to contract the social network of residents as they retreat into their homes and stop socializing on the street.

Gruenewald (2007) proposed in his Niche Theory that as outlet density increases, the competition for drinkers among the outlets in a neighborhood will lead to segmented drinking because the outlet owners appeal to various subsets of the drinking population (e.g., young drinkers versus old drinkers; gay bars versus straight bars). This theory is of particular relevance to the role of alcohol outlets in affecting sexual networks in the gay community. In neighborhoods with high alcohol outlet densities, it has long been observed that there is a segmentation of the gay community defined by the type of bar an individual frequents (e.g., leather bar versus pick up bar) (Achilles 1967). In addition, with segmentation comes a set of social and behavioral norms that define each bar, which typically includes behaviors associated with high risk of HIV transmission (e.g., high-risk sex, intravenous drug use). Niche Theory suggests that the social ecology associated with increasing alcohol outlet density will result in “hot spots” where risk of problem behavior will be excessively high (Gruenewald 2007).

### Societal Level

The societal level encompasses the policies that shape the neighborhood alcohol environment described above. For example, a number of studies have demonstrated higher densities of alcohol outlets (particularly liquor stores) in poor and minority neighborhoods (Gorman and Speer 1997; LaVeist and Wallace 2000; Romley et al. 2007) as well as a greater amount of shelf space dedicated to alcohol within these outlets (Bluthenthal et al. 2008). Theoretically, if a policy was enacted to reduce alcohol outlet density in a neighborhood, this would reduce the segmenting of drinkers. Assuming that the most segmented outlets are hot spots for HIV risk behaviors, HIV transmission would be reduced.

Scribner and colleagues (2008) evaluated the effect of a city-level policy designed to target alcohol outlet hot spots. In a sense, this research tests Gruenewald’s Niche Theory as it applies to HIV risk. In 1997, the city of New Orleans passed a series of ordinances to facilitate the prosecution of nuisance alcohol outlets. Over the years, the policy effectively eliminated problem alcohol outlets from a number of New Orleans neighborhoods. The researchers’ preliminary analyses revealed that annual risk of HIV/AIDS following the policy change decreased faster for individuals in New Orleans neighborhoods compared with Baton Rouge neighborhoods (Scribner et al. 2008).

## Discussion

The framework and conceptual model presented here suggest that exposures to alcohol and the alcohol environment play an important role in regulating the risk of acquiring HIV/AIDS. This effect is played out in the neighborhood and social contexts in which an individual is embedded, thereby regulating the risks to which they are exposed. Societal structures also play a role in shaping these neighborhood and social contexts. The policies affecting how alcohol is made available and the nature of the alcohol environment tend to reflect the social structure of greater society. For example, in a segregated city where Blacks tend to be governed by representatives of the White majority, the interests of the Black community may not be appreciated. In such a situation, formal and informal policies may result in situations where poorly run liquor outlets tend to proliferate in predominantly Black neighborhoods, thereby increasing situational risks for substance abuse and high-risk sexual behaviors. Poorly run alcohol outlets also influence the types of social and sexual networks that exist in a neighborhood and characteristics such as network density. Because Black minorities in a segregated society tend to be seen as “other,” the problems associated with a poorly run alcohol outlet are typically viewed as Black problems rather than problems associated with an alcohol outlet. In any case, exposure to these networks increases an individual’s risk of acquiring HIV/AIDs.

Demonstrating these types of relationships is part of the challenge in developing an ecological epidemiology for HIV/AIDS. Fortunately, a convergence of research methods and theory has made progress in this area possible. Ultimately, this approach holds promise for advancing efforts in HIV/AIDS prevention by addressing modifiable factors in the environment at the individual, interpersonal, neighborhood, and societal levels. ?
